# Discovery of 2D van der Waals layered MoSi_2_N_4_ family

**DOI:** 10.1093/nsr/nwaa190

**Published:** 2020-08-29

**Authors:** Kostya S Novoselov

**Affiliations:** Department of Materials Science and Engineering, National University of Singapore, Singapore; National Graphene Institute, University of Manchester, UK; Chongqing 2D Materials Institute, China

Two-dimensional (2D) materials have attracted increasing interest since the first isolation of graphene from graphite in 2004 [1] and subsequent exfoliation of other 2D crystals from a variety of layered, van der Waals (vdW), materials [2]. For such crystals, atoms within each layer are connected by strong chemical bonds, while the adjacent layers are stacked with weak vdW forces. Therefore monolayers can be exfoliated from the three-dimensional (3D) layered crystals, also called the parent materials. So far, dozens of such 2D vdW materials have been synthesized, such as graphene, *h*-BN, transition metal dichalcogenides and phosphorene [2–4]. The discovery of such 2D vdW materials has led to the observation of numerous exciting physical phenomena and exotic properties and already resulted in a number of intriguing applications. Furthermore, such 2D materials enable creation of a very broad class of artificial materials, vdW heterostructures, by layer-by-layer stacking in a designed sequence [3,4]. However, the structure of 2D vdW materials is essentially limited for those exfoliated from the parent materials. Synthesizing 2D vdW materials without known 3D layered parents would provide a huge opportunity for engineering materials with new attributes and functionality.

Of all known 3D materials, the majority are non-layered, including metals, transition metal carbides/nitrides (TMC/TMN), metal dicalcogenides, metal oxides, III-V semiconductors and organic-inorganic perovskites. Different from the vdW materials, the atoms in non-layered materials are connected by strong chemical bonding in 3D directions, and therefore their 2D forms cannot be obtained by direct exfoliation. Bottom-up growth is an alternative way to synthesize 2D non-layered materials [5,6], although it is very difficult to achieve continuous monolayer film because of the surface energy constraint [7]. Recently, a number of monolayer non-layered materials have been realized [6], such as silicene, germanene, bismuthene, tellurene and borophene, through use of a suitable substrate with strong adhesion to their atoms, which makes the atoms wet the substrate surface to realize 2D layer growth. However, such 2D materials are inherently not stable in ambient conditions because of unsaturated surface dangling bonds, which have been observed to oxidize when exposed to air.

**Figure 1.**
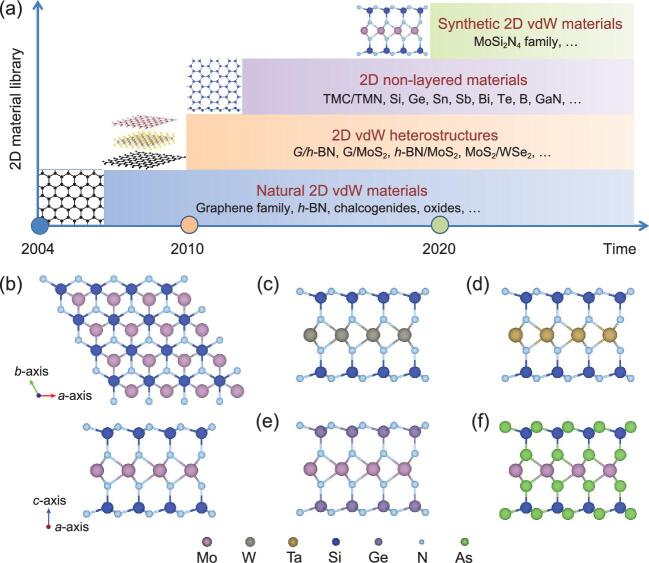
(a) Development of 2D material families. Natural 2D vdW materials refer to those having known analogous 3D layered allotropes in nature. G represents graphene. (b–f) Atomic structures of a few members of the 2D vdW layered MoSi_2_N_4_ family: (b) MoSi_2_N_4_, (c) WSi_2_N_4_, (d) TaSi_2_N_4_, (e) MoGe_2_N_4_ and (f) MoSi_2_As_4_.

In a recent work published in *Science* [8], a research team led by Wencai Ren from the Institute of Metal Research, Chinese Academy of Sciences, has made a significant breakthrough in synthesis of 2D vdW materials without known 3D parents (Fig. 1a). They proposed a new general concept to achieve such materials using appropriate elements to passivate the high-energy surfaces of non-layered materials during growth. Thus, they introduced elemental silicon during chemical vapor deposition growth of non-layered molybdenum nitride, which enabled growth of centimeter-scale monolayer films of a new vdW material, MoSi_2_N_4_ [8]. This monolayer was built up by septuple atomic layers of N-Si-N-Mo-N-Si-N (Fig. 1b), which can be viewed as a MoN_2_ layer sandwiched between two Si-N bilayers (the basic structural unit of Si_3_N_4_). It shows semiconducting behavior with potentially high carrier mobility as well as higher strength and better stability than most monolayer semiconductors such as MoS_2_. The successful growth of monolayer WSi_2_N_4_ using the same approach demonstrates the universality of the concept.

The growth of monolayer MoSi_2_N_4_ and WSi_2_N_4_ led to a new class of 2D vdW layered materials with the same structure and a formula MA_2_Z_4_ [8]. Their unique sandwich structure opens up possibilities to integrate diverse properties by rational design of the sandwiched building block and encapsulation with passivation layers. Density functional theory calculations were used to predict a large family of monolayer structured MA_2_Z_4_ materials with varying properties (Fig. 1b–f), where M represents an early transition metal (Mo, W, V, Nb, Ta, Ti, Zr, Hf or Cr), A is Si or Ge, and Z stands for N, P or As. For instance, monolayer WSi_2_N_4_ has a wider bandgap than monolayer MoSi_2_N_4_, whereas monolayer MoSi_2_As_4_ has a narrow direct bandgap in near-infrared range, and monolayer VSi_2_N_4_ is a half-metallic magnetic material.

The discovery of this 2D vdW layered MoSi_2_N_4_ family represents a milestone in development of 2D materials (Fig. 1a). Not only does it greatly expand the family of 2D materials, but, more importantly, it has opened up a new paradigm and research field of synthetic 2D vdW layered materials, which have no known 3D layered allotropes. Such 2D crystals can now be synthesized by passivating non-layered compounds with an appropriate passivation element. Besides the MA_2_Z_4_ materials presented in Ref. [8], this concept should also be applicable to other non-layered materials. This will greatly advance discovery of new 2D vdW layered materials with fascinating properties and intriguing applications that could not be achieved with existing 2D materials. Considering the very broad spectrum of properties of such 2D building blocks, many interesting physical phenomena such as superconductivity, topological order, 2D magnetism, valley polarization, excitonic effects and valley Hall effect could be observed. It is expected that such materials may find applications ranging from electronics, optoelectronics, photonics, spintronics, flexible devices, to membranes, composites, catalysis and energy conversion and storage.

***Conflict of interest statement***. None declared
